# Quality of life and informal care burden associated with duchenne muscular dystrophy in Portugal: the COIDUCH study

**DOI:** 10.1186/s12955-022-01941-x

**Published:** 2022-03-03

**Authors:** Valeska Andreozzi, Pedro Labisa, Melina Mota, Susana Monteiro, Rita Alves, João Almeida, Björn Vandewalle, Jorge Felix, Katharina Buesch, Hugo Canhão, Igor Beitia Ortiz de Zarate

**Affiliations:** 1 Exigo Consultores, Lisbon, Portugal; 2 PTC Therapeutics International, Steinhausen, Switzerland; 3 PTC Therapeutics Portugal, Sintra, Portugal

**Keywords:** Duchenne muscular dystrophy, Disease progression, Quality of life, Informal care burden

## Abstract

**Background:**

To describe the reduced health-related quality of life (HRQoL) of duchenne muscular dystrophy (DMD) patients and their caregiver burden and to present its relationship with disease progression.

**Methods:**

This cross-sectional study assessed patient HRQoL with the 3-level version of the EuroQol-5D (EQ-5D-3L) and caregiver burden with the Work Productivity and Activity Impairment: General Health questionnaire. DMD patients and their caregivers were identified through Portuguese Neuromuscular Association (APN).

**Results:**

A total of 46 DMD main caregivers, of eight ambulant and 38 non-ambulant patients, completed the questionnaires. Over half (58.7%) of all non-ambulant patients were on ventilation support, either full-time (15.2%) or non full-time (43.5%). Non-ambulant patients had a lower mean utility scores than ambulant patients (− 0.05 versus 0.51, *p* value < 0.001). Caregivers of non-ambulant patients reported a significant mean daily activity impairment as compared to caregivers of ambulant patients (68% versus 23%, *p* value < 0.001). Among non-ambulant patients, both utility scores and caregiver impairment appeared to deteriorate according to a higher need for ventilation support, however, these results were not statistically significant.

**Conclusions:**

These results emphasise the significant negative impact that DMD progression has on the patient HRQoL, as well as caregivers’ ability to conduct their daily activities. Therapeutic options that stop or slow the disease progression could have a beneficial impact for both patients and caregivers.

## Background

Duchenne Muscular Dystrophy (DMD) is a rare X-linked disorder which leads to the progressive degeneration of muscle tissues due to the lack or deficiency of the protein dystrophin [1]. First symptoms appear around the age of four, when diagnosis is typically established [2].

In the natural course of the disease, lower limb function is the first to be affected, and as patients age, muscular damage tends to accumulate, leading to an increasing level of symptom severity and lower function scores [3, 4]. Loss of ambulation occurs between the ages of 8 and 14 years [3] and precedes the development of other pulmonary and cardiac complications, which constitute the main causes of death [5].

There is no cure for DMD [1]. A combination of non-pharmacological treatments (physical therapy, surgery, ventilation support) and pharmacological treatments (glucocorticoids, angiotensin-converting-enzyme [ACE] inhibitors, beta-blockers and, in nonsense mutation DMD patients, ataluren) have helped delay disease progression and alleviate symptom severity [1, 6]. Altogether this multiapproach comprehensive care has contributed to a substantial increase in median life-expectancy at birth, which now reaches up to 40 years of age [7].

Improved medical care practices also had a positive impact on health-related quality of life (HRQoL). More advanced disease stages, characterized by the loss of ambulation or presence of ventilation support, have typically been associated with a lower HRQoL [8]. In addition to the direct benefits of delaying disease progression, such as loss of ambulation at a later age [1, 6], a better management of symptom severity allows patients to sustain a higher HRQoL for much longer than they otherwise would [9].

Caregiver burden is also a great concern to most families. Patients require a high number of informal care hours, which has a detrimental effect on the mental and physical health of caregivers, as well as on their day-to-day activities and professional life [10]. Yet, to our knowledge, there is a lack of data assessing daily caregiver activity impairment according to the disease stage of the patients under their care.

The COIDUCH study aimed to assess the cost of illness (COI), HRQoL of patients and the burden to caregivers associated with DMD, in Portugal. This paper reports on the HRQoL of patients and caregiver burden, and presents their relationship with disease progression.

## Methods

### Study design and data collection

The COIDUCH study was a cross-sectional study that included patients with DMD and their caregivers. To be included, patients were required to have a DMD diagnosis and be registered at the Portuguese Neuromuscular Association (APN). Any patients receiving experimental drugs or placebo in randomized clinical trials were excluded.

Face-to-face interviews were conducted by trained interviewers between June and August, 2019. After written informed consent, caregivers answered a customized questionnaire. The questionnaire collected patient and main caregiver data on demographic and clinical characteristics, i.e., age, sex, diagnosis date, ambulatory and assisted ventilation status, caregiver relationship to the patient, and informal care provided.

Patient HRQoL was assessed with the 3-level version of the EuroQol-5D (EQ-5D-3L) proxy version 2 instrument [11], in which caregivers were asked to rate how they thought the patients under their care would rate their own HRQoL, were they able to do so. Caregiver burden was measured with the Work Productivity and Activity Impairment: General Health (WPAI:GH) questionnaire [12], which assesses the impact health problems have on work productivity and regular daily activity.

All patients and caregivers were informed about the study objectives and data confidentiality.

### HRQoL and caregiver burden measures

The EQ-5D-3L instrument comprises a descriptive system based on five dimensions of health status: mobility, self-care, usual activities, pain/discomfort and anxiety/depression [13]. Each dimension in the descriptive system has three levels of response (no problems, moderate problems, or extreme problems). A validated translation of this instrument to Portuguese was used [13]. The EQ-5D-3L descriptive index responses were mapped into a single dimension HRQoL utility value ranging from full health (utility = 1), over death (utility = 0) to health states perceived as worse than death (utility < 0), using validated Portuguese population norms [14].

The WPAI:GH questionnaire [12] is a validated instrument and consists of six items covering employment status, hours missed from work due to health problems, hours missed from work due to other reasons, hours actually worked, how health problems affect productivity at work and how health problems affect regular daily activities. The questionnaire was adapted as to reflect the burden to the caregiver of the DMD patient under their care. The six questions were used to derive four domains: work time missed (absenteeism), impairment while working (presenteeism), overall work impairment (combining absenteeism and presenteeism) and activity impairment (daily activities). WPAI:GH outcomes on all four domains are expressed as impairment percentages (0–100%), with higher numbers indicating greater impairment and less productivity [15].

### Statistical analysis

Summary statistics were calculated for the overall population demographic, clinical, EQ-5D-3L and WPAI:GH data, as means and standard deviations (SD) for continuous data and absolute numbers and relative frequencies for categorical data.

Disease progression was evaluated according to the ambulatory status and/or ventilation support of patients. The tests of the null hypothesis of no difference on EQ-5D-3L utility and WPAI daily activity impairment according to ambulatory status and ventilation support were performed by the Wilcoxon and Kruskal–Wallis tests, respectively.

All analyses were performed using the R statistical software, considering a significance level of 5% [16].

## Results

A total of 53 patient/caregiver pairs identified by APN were invited to take part in the study. Of those, three did not meet the inclusion criteria and four declined to participate. Therefore, 46 patient/caregiver pairs met the inclusion criteria and were available to participate in the study.

All DMD patients were male, with a mean age of 18.9 (SD = 8.2) years. Of these, 38 (82.6%) were non-ambulant, having lost ambulation at a mean age of 9.7 (SD = 2.3) years. Over half (58.7%) of all non-ambulant patients were on ventilation support, either full-time (15.2%) or non full-time (43.5%).

The main caregivers reported a mean of 12.6 (SD = 5.6) hours of daily informal care and were mostly parents to the DMD patient (Table 1).

**Table 1:** Characteristics of the study participants

	Total cohort (N = 46)
Patients	
Mean age, years	18.9 ± 8.2
Mean age at diagnosis, years	4.4 ± 2.4
Non-ambulant patients, n (%)	38 (82.6)
Mean age at loss of ambulation, years	9.7 ± 2.3
Patients on assisted ventilation, n (%)	27 (58.7)
Full-time	7 (15.2)
Non full-time	20 (43.5)
Mean age at start of assisted ventilation, years	16.9 ± 4.3
Caregiver^a^	
Female, n (%)	39 (84.8)
Mean age, years	48.8 ± 9.8
Relationship to the patient, n (%)	
Parent	44 (95.7)
Other family member	2 (4.3)
Number of daily informal care hours	12.6 ± 5.6

Data presented as mean ± sd or n (%). Because of rounding, percentages might not add up to exactly 100%

^a^Only the main caregiver data was included

### Quality of life

Table 2 reports summary statistics on the main caregiver assessment of how patients would rate their own HRQoL. With exception to anxiety/depression, the majority of patients were rated as having either moderate or extreme problems in all EQ-5D-3L dimensions. The highest prevalence of extreme problems was in the self-care (78.3%) dimension, followed by mobility (37.0%) and usual activities (34.8%).

**Table 2:** Caregiver assessment of the patient 3-level response distribution of the five domains in the EQ-5D-3L questionnaire

	EQ-5D-3L dimensions (N = 46)				
	Mobility	Self-Care	Usual activities	Pain and discomfort	Anxiety and depression
No problems	1 (2.2)	4 (8.7)	5 (10.9)	15 (32.6)	26 (56.5)
Moderate problems	28 (60.9)	6 (13.0)	25 (54.3)	30 (65.2)	19 (41.3)
Extreme problems	17 (37.0)	36 (78.3)	16 (34.8)	1 (2.2)	1 (2.2)

Data presented as n (%); Because of rounding, percentages might not add up to exactly 100%

**Fig. 1:**
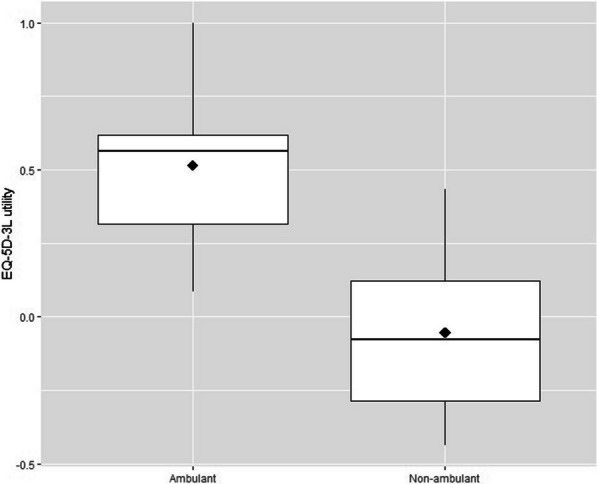
Box-plots of utility values in ambulant and non-ambulant patients. Diamond = mean value

Overall, DMD patients had a low HRQoL, with a mean utility value of 0.05 (SD = 0.33). The presence of negative utility values was higher among non-ambulant patients (61%), with a mean utility value of − 0.05 (SD = 0.24), while entirely absent in ambulant patients, which had a mean utility value of 0.51 (SD = 0.28). As shown in Fig. 1, the difference in utility values according to ambulatory status was statistically significant (*p* value < 0.001).

**Fig. 2:**
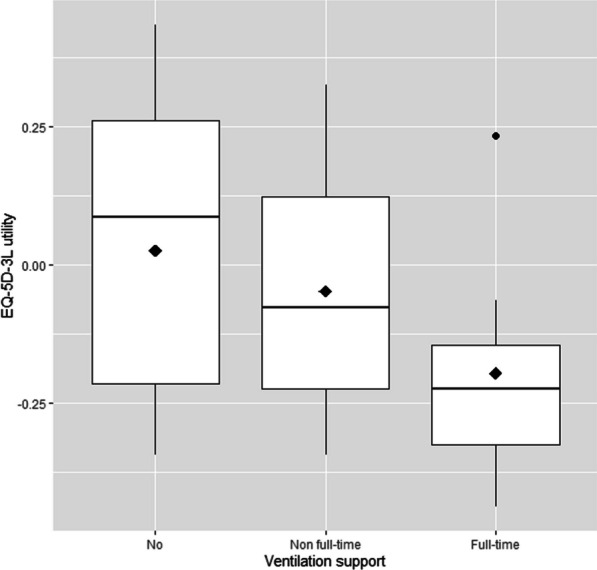
Box-plots of utility values according to ventilation support (no, non full-time and full-time) in non-ambulant patients. Diamond = mean value; dot = outlier

Figure 2 presents boxplots of the utility values and the mean utilities among non-ambulant patients according to ventilation support. Patients with full-time ventilation support presented the lowest mean utility value (− 0.20; SD = 0.22), followed by patients with non full-time ventilation support (− 0.05; SD = 0.21) and no ventilation support (0.03; SD = 0.29). Although data suggest a decline of HRQoL utility values while the need for ventilation support increases, statistical significance was not reached (*p* value = 0.228).

The presence of negative utilities was higher in full-time (86%) and non full-time ventilated (60%) patients, slightly less so in patients with no ventilation support (45%). There were, however, no statistically significant difference between groups (*p* value = 0.233).

### Work productivity and activity impairment

A total of 15 (32.6%) caregivers were employed at the time of interview. Of these, 12 had worked in the 7 days prior to the interview and were able to respond to the absenteeism and presenteeism related domains of the WPAI:GH questionnaire. Despite a low level of reported impact on absenteeism (11.1% in terms of work time missed), there was a relatively high level of impact of caring duties on presenteeism (35.8% impairment while working), leading to an overall level of work impairment of 30.5% (Table 3).

**Table 3:** Caregiver work productivity impairment (WPAI:GH) scores (%)

	Mean ± SD
Caregivers who worked in the past 7 days (n = 12)	
Percent work time missed due to patient’s DMD (absenteeism)	11.1 ± 13.1
Percent impairment while working due to patient’s DMD (presenteeism)	35.8 ± 22.8
Percent overall work impairment due to patient’s DMD (combining absenteeism and presenteeism)	30.5 ± 19.0
All caregivers (n = 46)	
Percent activity impairment due to patient’s DMD (daily activities)	60 ± 32.2

**Fig. 3:**
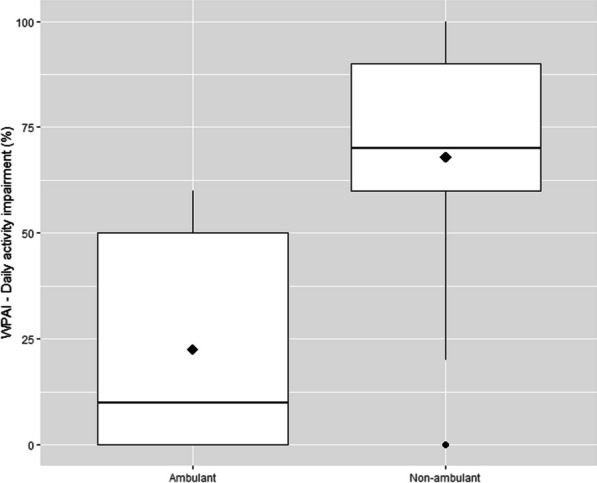
Box-plots of daily activity impairment in caregivers of ambulant and non-ambulant patients. Diamond = mean value; dot = outlier

Considering all 46 caregivers interviewed, the mean reported daily activity impairment was 60% (Table 3). Caregivers of non-ambulant patients reported a mean daily activity impairment of 68%, a value significantly higher as compared to caregivers of ambulant patients, which reported a mean value of 23% (*p* value < 0.001) (Fig. 3).

**Fig. 4:**
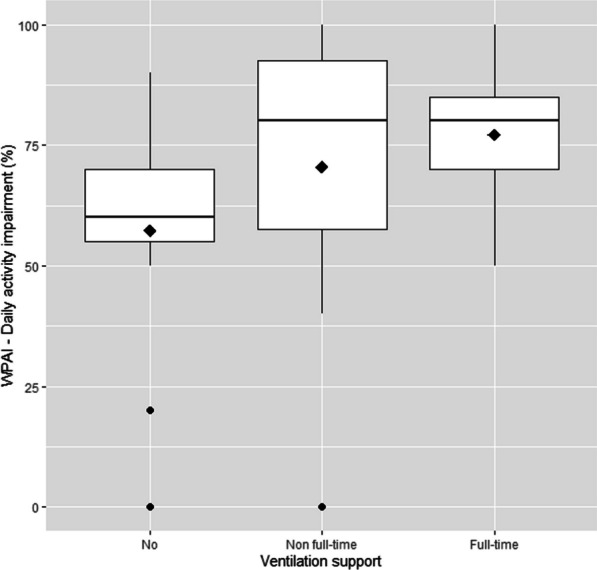
Box-plots of reported daily activity impairment of caregivers according to ventilation support (no, non full-time and full-time) in non-ambulant patients. Diamond = mean value; dot = outlier

Caregivers of non-ambulant patients with either full-time or non full-time ventilation support presented a higher daily activity impairment (77% and 71%, respectively), compared to patients without ventilation support (57%), but there were no statistically significant differences between groups (*p* value = 0.173) (Fig. 4).

## Discussion

To the best of our knowledge this was the first study to assess both DMD patient HRQoL and caregiver burden according to disease stage, measured by the EQ-5D-3L and WPAI:GH instruments, respectively. Given its known and predictable progression pathway, disease severity in DMD patients is usually categorized according to functional stages, related to ambulatory status, as well as other relevant clinical factors, such as the need for ventilation support.

Our results confirm that loss of ambulation is an important clinical event, that occurs early in life, around the age of 10, and is associated with a substantial impact in HRQoL. The mean utility in non-ambulant patients was − 0.05, significantly lower than that of ambulant patients (0.51). In non-ambulant patients there was also a trend towards lower utility values, as the need for ventilation support, expressed by frequency of use, increased. However, this trend did not prove to be statistically significant.

These results are consistent with those in other studies which have shown lower health utility values associated with non-ambulation, as well as more advanced disease stages, such as full-time ventilation support [17–20]. One distinct feature, however, relates to the presence of negative mean utility values in non-ambulant patients, suggesting that these patients may be seen by society as being in a health state worse than death. This diverges from most studies which have suggested low, but positive, utility values in DMD patients, including those in late-stage disease [8]. One exception being Cavazza et al. [18], in which adult DMD patients from Spain, Sweden and United Kingdom reported negative utilities, while also using the EQ-5D-3L questionnaire.

One possible explanation resides in the use of different country specific value sets, making this heterogeneity in utilities a reflection of different societal perceptions for the same health states. However, it remains unclear to which extent other factors, such as patient heterogeneity or perspective used to assess the patients’ HRQoL (patient vs caregiver reporting), may have contributed to this discrepancy in results.

Our results also show that disease progression has a substantial impact on caregiver burden, assessed by the percentage of caregiver daily activity impairment. Caregivers of non-ambulant patients reported a mean daily activity impairment of 68%, close to three times that of caregivers of ambulant patients (23%), with a statistically significant difference between both groups (*p* value < 0.001). There was also a trend towards higher impairment levels in caregivers of non-ambulant patients with higher need for ventilation support, but this trend did not reach statistical significance. As disease severity increases, caregivers allocate an increasing percentage of their time to informal care, which explains the high number of daily informal care hours reported (12.6). This not only limits their ability to retain employment, but also reduces the number of leisure hours left in the day, and is consistent with the results in other published studies, albeit with the use of alternative questionaries [3, 10].

The cross-sectional data presented helps understand the consequences that disease progression carries for important patient and caregiver outcomes. Current treatment options, such as corticosteroids, have shown to delay the natural course of disease progression, namely loss of ambulation and respiratory function, and their use is recommended in ambulant patients and, to a lesser extent, in non-ambulant patients as well [1]. However, the results from our study show that despite the potential benefits of the current treatment options, disease progression and its direct consequences, both in terms of utility deterioration and caregiver daily activity impairment, are still a major source of concern. Recent innovative treatment options, such as ataluren, have shown to further delay disease progression in certain groups of patients (nonsense mutation DMD), including important clinical outcomes such as loss of ambulation or pulmonary function [21]. This delay in critical outcomes will likely allow patients to maintain higher utility levels for longer periods of time, as well as decrease caregiver burden. Hence, ensuring conditions for adequate access to innovative treatments for DMD patients appears to be of critical importance.

Moreover, addressing caregiver burden through other measures, such as expanding the financial and social support schemes available to affected families, should also be considered. The recent introduction of a formal status for informal caregivers in Portugal, which aims to improve financial support, caregiving training, psychological support and help reconcile patient care duties with professional life, represents an important step forward [22].

In addition to the use of two validated questionnaires which had not been used before in conjunction in this context, another asset of this study resides in the way data was collected, with face-to-face interviews, which helped minimize the risk of questions being misinterpreted, at least when compared to other more indirect methods.

One limitation of this study relates to the potential for referral bias from having the recruitment process of DMD patients relying on a prior membership in the APN. Membership in this association is voluntary and may be more attractive for patients with a more severe clinical presentation of DMD, since it is attached with a greater availability of clinically relevant services, such as rehabilitation.

Another limitation relates to the sample size of patients included in this study. Even though our sample likely represents a large percentage of the expected prevalence of DMD in Portugal, the total number of patients included was still smaller compared to that in other studies [10, 17, 18, 23]. This may have contributed to the lack of statistical significance in some of the comparisons performed, such as in non-ambulant patients according to the need for ventilation support.

To ensure that the type of HRQoL data was consistent between paediatric and adult patients, EQ-5D-3L was assessed exclusively through the use of caregiver-proxy reporting. Studies have shown that for the same health states, utility data obtained from proxy reporting tends to be lower than that of patient reporting [8]. This is a limitation that may help further explain the high presence of negative utility values among non-ambulant patients in our study.

In addition to the use of a WPAI:GH questionnaire adaptation, which was not validated for the use in DMD, our exclusive focus on main caregiver burden likely resulted in an underestimation of the total caregiver burden, since it neglected the contribution of other family members to the informal care of the patient.

## Conclusion

In conclusion, our results emphasise the impact of DMD on the HRQoL of patients and the burden to their caregivers, which gets increasingly detrimental as disease progresses. This highlights the need for therapeutic options that can stop or slow down disease progression, at any point in the disease history.

## Data Availability

All aggregate data generated or analysed are included in this published article. No individual data is available to protect the recognition of individual patients.

## Abbreviations

ACE: Angiotensin-converting-enzyme
APN: Portuguese Neuromuscular Association
COI: Cost of illness
DMD: Duchenne muscular dystrophy
EQ-5D-3L: 3-Level version of the EuroQol-5D
GDPR: General Data Protection Regulation
HRQoL: Health-related quality of life
SD: Standard deviation
WPAI:GH: Work Productivity and Activity Impairment: General Health
